# Analysis of risk factors affecting the postoperative drainage after a laparoscopic partial nephrectomy: a retrospective study

**DOI:** 10.3389/fmed.2024.1327882

**Published:** 2024-01-24

**Authors:** Yi-Qun Tian, Xiang Ren, Yi-Sheng Yin, Jing Wang, Xing Li, Zi-Hao Guo, Xiao-Yong Zeng

**Affiliations:** ^1^Department of Urology, Tongji Hospital, Tongji Medical College, Huazhong University of Science and Technology, Wuhan, China; ^2^China Institute of Urology of Hubei Province, Wuhan, Hubei, China

**Keywords:** laparoscopic partial nephrectomy, smoking history, diabetes, BMI, age

## Abstract

**Purpose:**

Laparoscopic partial nephrectomy (LPN) remains the most commonly used measure for treating localized renal cell cancer (RCC) with an increasing incidence of RCC ever since the 1990s. This study aimed to identify risk factors that affect the postoperative time of drainage and total drainage volume after LPN.

**Method:**

The clinical data of 612 RCC patients who received LPN from January 2012 to December 2022 in our hospital, including the postoperative drainage time and total drainage volume, were retrospectively analyzed. Univariable and multivariable linear regression and correlation analyses were used to identify the correlations between 21 factors, which include gender, age, history of alcohol consumption, family history of RCC, body weight, body mass index (BMI), and operation time, postoperative drainage time, and total drainage volume.

**Results:**

The mean time of drainage was 3.52 ± 0.71 days (range: 2 to 8 days), with an average total drainage volume of 259.83 ± 72.64 mL (range: 50 to 620 mL). Both univariable and multivariable linear regression analyses revealed several statistically significant associations. Gender (*p* = 0.04), age (*p* = 0.008), smoking history (*p* < 0.001), diabetes (*p* = 0.032), operation time (*p* = 0.014), and BMI (*p* = 0.023) were identified as significant factors associated with the time of drainage. On the other hand, age (*p* = 0.008), smoking history (*p* < 0.001), diabetes (*p* = 0.006), and BMI (*p* = 0.016) emerged as independent risk factors influencing the total drainage volume.

**Conclusion:**

The duration of postoperative drainage was found to be associated with gender, age, smoking history, diabetes, operation time, and BMI. In contrast, the total drainage volume was primarily influenced by age, smoking history, diabetes, and high BMI following LPN. For patients with these conditions, meticulous attention to hemostasis and bleeding control is crucial during the perioperative period.

## 1 Introduction

For decades, the development and widespread use of imaging technology as well as the improvement of people’s health awareness aroused the increasing incidence of localized renal cell cancer (RCC) year by year (1), according to the latest data from the International Agency for Research on Cancer, the incidence of kidney cancer ranked ninth among men (2). Partial nephrectomy has gained widespread acceptance among experts worldwide as the preferred treatment for localized RCC. It is considered the gold standard due to its ability to reduce postoperative mortality, preserve renal function, and achieve comparable surgical outcomes. Among various surgical methods, laparoscopic partial nephrectomy (LPN) is currently the most commonly utilized approach (3, 4). Due to the kidney’s high vascular perfusion, effective postoperative drainage is essential in the care of patients who have undergone LPN. This drainage facilitates the efficient removal of excess fluid, blood, and potential contaminants from the surgical site, thereby reducing the risk of complications such as hematoma formation, infection, and seroma formation. Additionally, monitoring the drainage volume provides valuable insights into the patient’s postoperative recovery, with abrupt changes in drainage volume serving as early indicators of potential complications, including bleeding or impaired wound healing. Consequently, urologists worldwide recognize the importance of postoperative drainage as a standard practice, contributing to shortened hospital stays and minimized risks of postoperative complications following LPN (5). However, a routine postoperative drainage indwelling may not be rational for all patients who have undergone LPN, as postoperative morbidity may be caused by drain-related complications, such as infections, hemorrhage, and damage to perinephric tissues (5, 6). Hence, whether a routine postoperative drainage indwelling remains necessary for all patients after LPN might be an essential conundrum for urologists.

As far as we know, no previous research has been conducted to identify the risk factors for postoperative drainage, including total drainage volume and drainage time following LPN. Therefore, we initiated this study to assess the factors influencing postoperative drainage and to establish criteria for selecting patients who will benefit from postoperative perinephric drainage.

## 2 Methods

### 2.1 Patients and data resource

After approval by the Medical Ethics Committee of Tongji Hospital, Tongji Medical College of Huazhong University of Science and Technology, a retrospective study was conducted, and this study was conducted in accordance with the Declaration of Helsinki. In addition, written informed consent was provided by all participants prior to this study.

**Figure 1:**
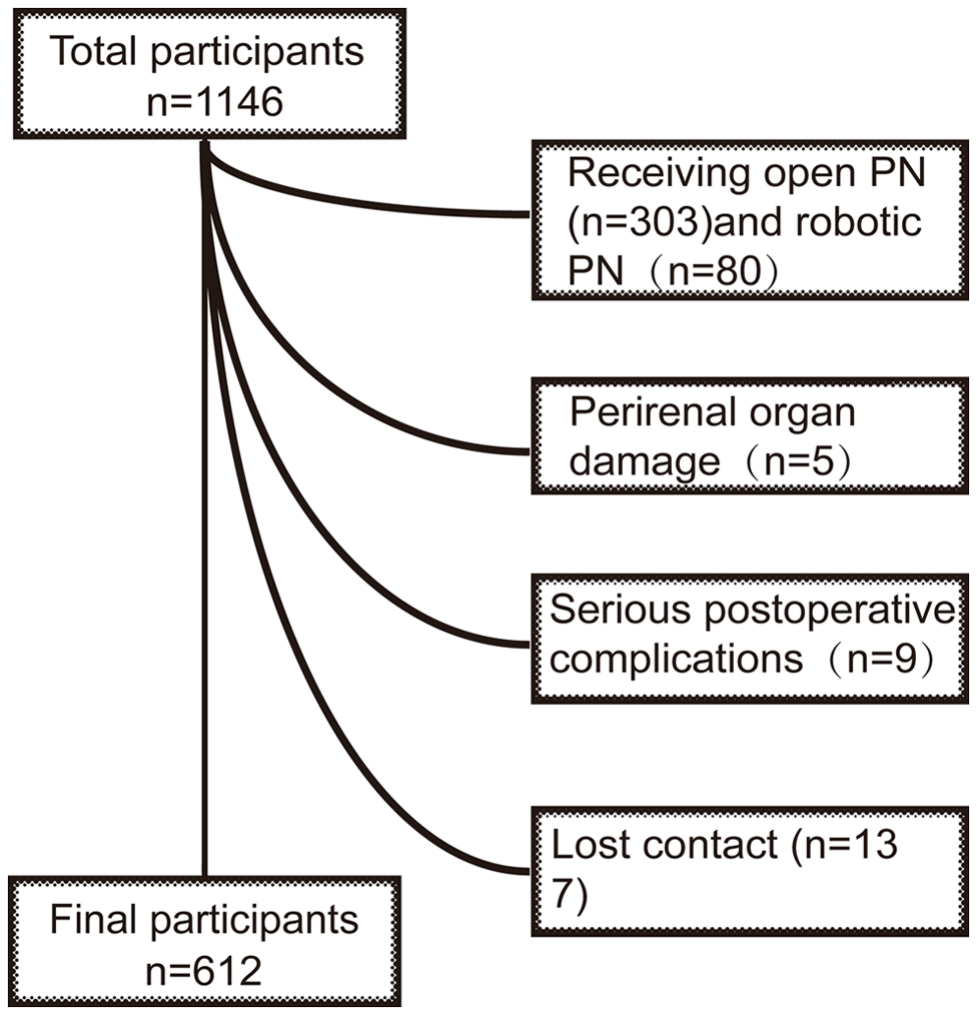
Flow of study participants in the study. After excluding 534 participants who received open PN (*n* = 303) or robotic PN (*n* = 80), suffering from perirenal organ damage (*n* = 5), or serious postoperative complications (*n* = 9), and lost contact (*n* = 137), those who completed a follow-up were recruited (*n* = 612). PN, partial nephrectomy.

A retrospective analysis of 612 patients who were diagnosed with RCC and received LPN by the same surgeon in our institution from January 2012 to December 2022 was conducted. The inclusion criteria were as follows: (1) preoperative estimation of tumor diameter ≤ 4 cm; (2) exclusion of distant metastasis of RCC and lymph node metastasis with chest CT and abdominal CT; (3) localized RCC confirmed by pathology; and (4) first surgery for RCC (patients with a previous history of simple renal cysts or renal angiomyolipoma were not excluded). The criteria for exclusion were as follows: (1) serious postoperative complications, including severe infection, hemorrhage, and urinary leak; (2) receiving reoperation due to perinephric organ damage; and (3) turning into open partial nephrectomy because of complicated conditions (Figure 1).

All laparoscopic partial nephrectomies in our study were performed by the same skilled surgeon at our hospital, following procedures and settings that had been previously described in a published article (7). In summary, we utilized a retroperitoneal approach for the majority of patients undergoing LPN in our study. To ensure optimal preservation of renal function while achieving a bloodless surgical field, warm ischemia with an ischemia time of less than 25 min was employed during LPN. In our research, drainage was measured in milliliters and specifically referred to as the total output solely from wound ooze, starting from the time of surgery until drain removal. We adopted a criterion of drain removal when the drainage volume remained below 50 mL for the previous 24 h to ensure no significant postoperative drainage was overlooked.

We collected the following data from all patients who participated in the study: age, gender, height, weight, body mass index (BMI), smoking history; hypertension, diabetes, heart disease, tumor diameter, tumor side, preoperative activated partial thromboplastin time (APTT), preoperative thrombin time (TT), preoperative blood protein, operation time, blood loss during operation, total drainage volume, and total days of drainage. Total drainage volume was measured in milliliters and calculated as the summation of the daily drainage volume from the first day of operation to the day of drainage removal; in addition, we define obesity and overweight according to the World Health Organization (WHO), a BMI of ≥25 is considered overweight, while a BMI of ≥30 means obesity (8).

### 2.2 Data analysis and statistics

For continuous variables, we presented the median (range) for non-normally distributed data and mean ± standard deviation (SD) for normally distributed data. Categorical variables were reported as absolute numbers and proportions. Univariate analyses included the use of Student’s *t*-test or the Mann–Whitney *U*-test for continuous variables and the chi-square test or Fisher’s exact test for categorical variables. To evaluate the relationship between postoperative drainage and independent patient variables, we conducted a univariate linear regression analysis. Variables demonstrating a significant univariate relationship (*p* < 0.05) were included in the subsequent multivariate linear regression analysis with postoperative drainage as the outcome variable. Linear correlations between variables were assessed using the Pearson correlation coefficient (Pearson’s r) for normally distributed variables or Spearman’s rank correlation coefficient (Spearman’s rho) for non-normally distributed variables. All statistical analyses were performed using SPSS 26.0 (SPSS Inc., Chicago, IL). Variables with a value of *p* less than or equal to 0.05 in the final model were considered significant. All value of *p*s were two-sided.

## 3 Results

### 3.1 General data

The study comprised 612 RCC patients who underwent LPN between January 2012 and December 2022, meeting the inclusive criteria. Of the included patients, 378 were men and 234 were women, with a mean age of 54.3 ± 16.28 years and a mean BMI of 25.24 ± 4.13 kg/m^2^. Among the patients, 37.2% (*n* = 228) were active smokers, 21.2% (*n* = 130) had a history of alcohol, 1.1% (*n* = 7) had a family history of RCC, while 69 (11.3%) had hypertension, 99 (16.2%) had diabetes, and 74 (12.1%) had heart diseases before operation. The average height and weight of the patients were 164.62 ± 5.74 cm and 66.72 ± 11.26 kg, respectively. Tumors were mostly observed in the left kidney (*n* = 409), with a mean diameter of 27.31 ± 10.21 mm. Additionally, preoperative blood protein, APTT, PT, and D-dimer had an average value of 66.71 ± 4.25 g/L, 36.82 ± 2.23 s, 16.82 ± 7.26 s, and 0.41 ± 0.33 ng/mL, respectively. During the operation, the mean blood loss was 95.92 ± 10.26 mL, and the mean operation time was 128.73 ± 31.2 min. Overall, the mean drainage time and average total drainage volume were 3.52 ± 0.71 days (range: 2 to 8 days) and 259.83 ± 72.64 mL (range: 50 to 620 mL), respectively (Table 1). More specifically, in men, the average drainage time and total drainage volume were 4.05 ± 1.68 days and 282.83 ± 79.73 mL, respectively. On the other hand, women had an average drainage time of 3.23 ± 1.33 days and a total drainage volume of 242.79 ± 68.57 mL (Supplementary Tables S1, S5). Our findings align with those of a prior publication that focused on East Asian women in Japan, which reported an average drainage removal time of 4 days and an average total drainage volume of 214 mL (7).

**Table 1:** Demographic and clinical characteristics of patients undergoing laparoscopic partial nephrectomy (*n* = 612).

Variables	Mean ± SD (range)	*n* (%)
Gender (male)	**-**	378 (61.8%)
Age (years)	54.3 ± 16.28	-
Height (cm)	164.62 ± 5.74	-
Weight (kg)	66.72 ± 11.26	-
BMI (kg/m^2^)	25.24 ± 4.13	-
Smoking history	-	228 (37.2%)
History of alcohol consumption	-	130 (21.2%)
Family history of RCC	-	7 (1.1%)
Hypertension	-	69 (11.3%)
Diabetes	-	99 (16.2%)
Heart diseases	-	74 (12.1%)
Tumor diameter (mm)	27.31 ± 10.21	-
Tumor side (left)	-	409 (66.9%)
Preoperative blood protein (g/L)	66.71 ± 4.25	-
Preoperative APTT (s)	36.82 ± 2.23	-
Preoperative PT (s)	16.82 ± 7.26	-
Preoperative D-dimer (ng/mL)	0.41 ± 0.33	-
Blood loss during operation (mL)	95.92 ± 10.26	-
Operation time (min)	128.73 ± 31.2	-
Time of drainage (day)	3.52 ± 0.71	-
Total drainage volume (mL)	259.83 ± 72.64	-

BMI, body mass index; APTT, activated partial thromboplastin time; PT, thrombin time; SD, standard error; RCC, renal cell cancer.

### 3.2 Univariable and multivariable linear regression analyses of risk factors influencing time of drainage and total drainage volume

Univariable and multivariable linear regression analyses were conducted to identify risk factors affecting the time of drainage as presented in Table 2. Among the variables analyzed, gender (*p* = 0.023), age (*p* = 0.010), smoking history (*p* < 0.001), hypertension (0.007), diabetes (*p* < 0.001), operation time (*p* < 0.001), height (*p* = 0.009), weight (*p* < 0.001), and BMI (*p* < 0.001) showed significant associations with the time of drainage in the univariable analysis. Subsequently, these nine variables were included in the multivariable analysis, and only gender (*p* = 0.04), age (*p* = 0.008), smoking history (*p* < 0.001), diabetes (*p* = 0.032), operation time (*p* = 0.014), and BMI (*p* = 0.023) remained statistically significant predictors of the time of drainage. Similar findings were observed when performing separate analyses for male and female patients, except for the correlation between operation time and time of drainage in female patients. While the univariable analysis suggested a potential relationship between operation time and time of drainage, the multivariable analysis did not support this correlation (Supplementary Tables S2, S6).

**Table 2:** Univariable and multivariable linear regression analyses of factors influencing the time of drainage (dependent variable; *n* = 612).

	Univariable			Multivariable		
	*β*	SE	Value of *p*	*β*	SE	Value of *p*
Gender	−0.213	0.097	0.023	0.342	0.153	0.04
Age	0.015	0.007	0.010	0.012	0.007	0.008
Smoking history	0.932	0.105	*p* < 0.001	0.518	0.137	*p* < 0.001
History of alcohol consumption	0.582	0.172	0.803	-	-	-
Hypertension	0.473	0.132	0.007	-	-	-
Diabetes	0.618	0.143	*p* < 0.001	0.241	0.097	0.032
Heart diseases	0.164	0.186	0.387	-	-	-
Operation time	0.004	0.001	*p* < 0.001	0.007	0.010	0.014
Tumor diameter	0.003	0.007	0.708	-	-	-
Tumor side	−0.10	0.154	0.793	-	-	-
Preoperative APTT	0.018	0.054	0.524	-	-	-
Preoperative PT	−0.002	0.006	0.624	-	-	-
Preoperative D-dimer	0.142	0.090	0.412	-	-	-
Blood loss during operation	0.008	0.002	0.371	-	-	-
Preoperative blood protein	−0.012	0.031	0.501	-	-	-
Height	0.042	0.013	0.009	-	-	-
Weight	0.051	0.015	*p* < 0.001	-	-	
BMI	0.162	0.007	*p* < 0.001	0.193	0.062	0.023

BMI, body mass index; APTT, activated partial thromboplastin time; PT, thrombin time; SE, standard error.

The association between risk factors and the total drainage volume was examined through univariable linear regression analyses. Among the seven risk factors evaluated, age (*p* = 0.001), smoking history (*p* < 0.001), diabetes (*p* = 0.012), operation time (*p* = 0.043), height (*p* = 0.010), weight (*p* < 0.001), and BMI (*p* < 0.001) showed significant associations with the total drainage volume. However, in the multivariable regression analysis, only age (*p* = 0.008), smoking history (*p* < 0.001), diabetes (*p* = 0.006), and BMI (*p* = 0.016) remained as significant risk factors influencing the total drainage volume (Table 3). Similar results were observed when conducting separate analyses for male and female patients, indicating that the identified risk factors remained consistent across genders (Supplementary Tables S3, S7).

**Table 3:** Univariable and multivariable linear regression analyses of factors influencing the total volume of drainage (dependent variable; *n* = 612).

	Univariable			Multivariable		
	*β*	SE	Value of *p*	*β*	SE	Value of *p*
Gender	−59.413	42.16	0.068	-	-	-
Age	3.587	1.013	0.001	2.415	0.872	0.008
Smoking history	158.748	29.532	*p* < 0.001	128.463	30.540	*p* < 0.001
History of alcohol consumption	97.283	43.633	0.826	-	-	-
Hypertension	43.672	32.753	0.226	-	-	-
Diabetes	141.465	41.52	0.012	46.371	30.660	0.006
Heart diseases	5.792	33.589	0.693	-	-	-
Operation time	2.313	0.822	0.043		-	-
Tumor diameter	2.216	1.392	0.624	-	-	-
Tumor side	17.311	29.486	0.713	-	-	-
Preoperative APTT	8.215	5.267	0.422	-	-	-
Preoperative PT	−1.382	1.964	0.801	-	-	-
Preoperative D-dimer	17.373	28.129	0.677	-	-	-
Blood loss during operation	0.031	1.087	0.842	-	-	-
Preoperative blood protein	−1.047	3.771	0.612	-	-	-
Height	7.331	3.378	0.010	-	-	-
Weight	13.98	0.902	*p* < 0.001	-	-	
BMI	33.192	4.037	*p* < 0.001	24.479	14.533	0.016

BMI, body mass index; APTT, activated partial thromboplastin time; PT, thrombin time; SE, standard error.

### 3.3 Pearson correlation and Spearman’s rank correlation analyses between other studied variables except for the time of drainage and total drainage volume

Pearson correlation coefficient and Spearman’s rank correlation were utilized to evaluate the linear correlation between the analyzed variables. Our study found that women had lower average age, smoking history, hypertension, and BMI than men. However, there was no significant difference in diabetes, operation time, and tumor diameter between the two genders. Interestingly, older patients had a higher rate of smoking history than younger patients. The results of correlation analyses in men and women were all similar to those in both sexes; generally, smoking history was positively correlated with hypertension and operation time, while a larger tumor diameter was significantly associated with longer operation time. Additionally, BMI showed several positive correlations, with gender, history of alcohol consumption, hypertension, diabetes, and operation time being among the variables that demonstrated statistically significant correlations (*p* < 0.05) (Table 4; Supplementary Tables S4, S8).

**Table 4:** Pearson correlation and Spearman’s rank correlation analyses between other studied variables except for time of drainage and total drainage volume.

	Gender	Age	Smoking history	History of alcohol consumption	Hypertension	Diabetes	Heart diseases	Operation time	Tumor diameter	BMI
Gender	1	-	-	-	-	-	-	-	-	-
Age	−0.203*	1	-	-	-	-	-	-	-	-
Smoking history	−0.322**	0.331**	1	-	-	-	-	-	-	-
History of alcohol consumption	−0.268*	0.227*	0.349*	1	-	-	-	-	-	-
Hypertension	−0.125**	0.073	0.221*	0.186*	1	-	-	-	-	-
Diabetes	0.013	0.068	0.022	0.087	0.026	1	-	-	-	-
Heart diseases	0.007	0.063	0.09	0.253	0.05	0.008	1	-	-	-
Operation time	−0.031	0.042	0.093*	0.304	0.04	0.087	0.05	1	-	-
Tumor diameter	0.012	−0.08	0.071	0.086	−0.036	−0.042	0.024	0.147**	1	-
BMI	−0.266**	−0.046	0.482	0.207*	0.148**	0.283**	0.031	0.186**	−0.023	1

*Correlation is significant at the 0.05 level (two-tailed); **Correlation is significant at the 0.01 level (two-tailed). BMI, body mass index.

## 4 Discussion

Although LPN remains the most commonly performed surgical method for treating localized RCC today, the etiology and risk factors determining the postoperative drainage perseveres unclear (3–5). Our study was the first study to seek to identify risk factors for increased postoperative drainage in patients having undergone LPN, and we found that gender, smoking history, diabetes, operation time, and BMI were independent risk factors for the time of drainage by univariable and multivariable linear regression analyses; meanwhile, age, smoking history, diabetes, and BMI showed a significant association with total drainage volume after LPN.

Our findings were consistent with other studies, which also identified a significant positive correlation between age and both the time of drainage and total drainage volume. These results highlight the potential impact of age on postoperative recovery and emphasize the need for careful monitoring and management in elderly patients undergoing surgery (9, 10). Lee et al. (11) also reported that advanced age was an independent risk factor with increased time of drainage. Age could be a significant factor influencing postoperative drainage due to the varied progression of wound healing in the elderly population. The aging process may contribute to a higher risk of postoperative complications, leading to potential issues with wound healing. Following a cutaneous injury, the wound healing response depends on an intricate interplay of biochemical and mechanical signals, coordinating the phases of hemostasis, inflammation, proliferation, and remodeling. This sequential process is crucial for the successful healing of a wound (12). However, it was generally accepted that aging skin wounds heal inversely compared with younger wounds, which are associated with delayed proportions of closure and mechanically frailer tissue following repair (13). Ashcroft et al. (14) demonstrated a 4-day delay of leukocyte concentration and ensuing inflammatory phase, regardless of a prolonged inflammatory phase of wound healing in aging murine model than in young mice. Furthermore, aging skin exhibits reduced glycosaminoglycan content, delayed proliferation and migration, and decreased fibrosis. Additionally, altered collagen fiber remodeling and increased stiffness are commonly observed due to an increase in cell senescence, leading to heightened fibrinogen polymerization and tissue repair hardness, ultimately resulting in disrupted wound healing.

Our results revealed that smoking was significantly associated with increased time of drainage as well as drainage volume, which were comparable with that of other studies investigating risk factors for postoperative drainage (15, 16). Previous studies have demonstrated that smoking remains the leading risk factor attributable to the incidence and mortality of RCC and has a modifiable effect on patients who have undergone surgeries (17, 18). It is generally acknowledged that smoking was a noteworthy risk factor for delayed wound healing and improved chance of infection (19), which contributes to increased postoperative drainage after surgeries; Moller et al. (20) had reported that a smoking history had been majorly associated with delayed wound healing, and patients with a smoking history had a 2.68 relative risk of increased drainage compared with those without a history of smoking (*p* = 0.044) in this research; and Tomoyoshi et al. also demonstrated that smokers posed a 1.45 relative risk of increased drainage volume (*p* = 0.04) and a 1.18 times increment in time of drainage compared with never smokers. A meta-analysis (17) demonstrated that smoking suppresses the inflammatory healing response, decreases local tissue oxygenation, and downregulates proteolytic and synthetic enzyme activity to affect the progress of wound healing. Additionally, preoperative smoking cessation has been reported to be associated with decreased postoperative wound-related complications after surgeries (16, 21–25).

Canbek et al. (26) reported that compared with patients with a lower BMI, those with a BMI ≥40 had a significant prolonged time of drainage. Inoue et al. (27) found that patients with BMI had a higher total drainage volume and longer time of drainage. Long et al. (28) reported a positive correlation between BMI and time of drainage and total drainage volume, and Lee et al. (11) also reported a significant positive correlation between BMI and drainage time. These above results are supported by that of the present study which identified that higher BMI was significantly associated with higher total drainage volume and longer time of drainage. Furthermore, our previous study (18) demonstrated that the kidney cancer mortality rate attributable to high BMI had been increasing and persists as the leading risk factor for female kidney cancer deaths since 1999. In 2019, the contribution of a high BMI to kidney cancer death even exceeded that of smoking, especially in advanced countries and regions due to high income levels and high-fat eating habits.

In this study, patients with diabetes suffered a longer time of drainage and higher total drainage volume and univariable and multivariable linear regression analyses revealed that diabetes was significantly positively correlated with time of drainage and total drainage volume. Previous studies had demonstrated that diabetic patients were associated with a high rate of postoperative complications, including longer hospital stays, increased postoperative infection, and mortality (29). Steve et al. (30) reported an increasing risk of infection for every 10-unit increase in the highest glucose, while Chen et al. (31) advised that hyperglycemic conditions increased the total drainage volume, which was consistent with our study. Perioperative measures such as the application of insulin or hypoglycemic drugs are also recommended to reduce the incidence of postoperative complications.

Preoperative surgical planning and intraoperative technique were all important influencing factors of surgical outcomes, and the selection of the above content had always been the focus of clinicians’ attention. For example, in our study, we followed the Chinese Urology Association Guideline 2022 and our own experience by limiting the renal artery clamp time to a maximum of 25 min during LPN. This approach aimed to minimize renal acidosis and preserve renal function as much as possible. However, there remains controversy regarding the impact of warm ischemia time (WIT) on long-term renal function recovery, and Abdel et al. (32) demonstrated that the duration of WIT during LPN does not affect long-term renal function outcomes. Furthermore, the potential impact of WIT on postoperative drainage has not been established yet. Thus, future research focusing on the correlation between these two variables may provide valuable insights into the influence of WIT on patient outcomes. Moreover, the surgical approach used during LPN was crucial for both intraoperative procedures and postoperative recovery of patients as it can affect the extent of anatomy damage during the procedure and the length of recovery. Compared to the transperitoneal approach, the retroperitoneal approach offers similar postoperative, functional, and oncological outcomes, but with a shorter operative time (33), which was observed positively correlated with operative time in our research. As such, we hypothesized that the retroperitoneal approach may result in less drainage time. However, the relationship between surgical approach and drainage had yet to be established, and further research was needed to draw a definitive conclusion. Finally, as urologists had advanced their understanding of LPN techniques, the off-clamp approach, which aims to minimize or eliminate renal ischemia during the procedure, gained attention. Bertolo et al. (34) reported comparable perioperative and early functional outcomes between off-clamp and on-clamp LPNs. It was worth noting that all LPNs in our study were performed using the on-clamp technique, and the potential impact of on- or off-clamp approaches had yet to be determined. Future prospective research was needed to further investigate and identify any differences between these two techniques.

Our study was pioneering in identifying the risk factors associated with postoperative drainage and establishing criteria for selecting patients who would benefit from perinephric drainage after surgery. This valuable information provides surgeons with guidance on the necessity of routine drainage indwelling, helping them avoid potential complications related to unnecessary drainage. In our study, we found that gender, age, smoking history, diabetes, operation time, and BMI were significantly associated with the duration of drainage. Moreover, elderly patients, those with a history of smoking, diabetes, and higher BMI are at a greater risk of having a larger total drainage volume, suggesting that patients with these factors may benefit from drainage indwelling to a greater extent. In addition, these operations were completed by the same surgeon, with the same technique and procedures in the current study, thereby minimizing bias caused by the habits of the operator as adequately as possible. There are two limitations of our study; first of all, the sample size of our study was small and a greater clinical significance would be exhibited by this study if a larger sample size was involved. On the other hand, only one experienced surgeon was involved in this study; data sources from multiple centers and other experienced doctors are needed to verify our conclusions. Moreover, due to the retrospective nature of this study, only association, rather not causation, between the above risk factors and the examined outcomes can be definitively concluded. In addition, we assume that for patients without these conditions, a routine drainage indwelling may not be recommended as more damage rather than benefit might be instigated. However, based on the retrospective nature of this study, a further prospective controlled study was required to determine whether patients without risk factors can be exempted from routine drainage.

## 5 Conclusion

The necessity of routine drainage following LPN had not been definitively established. Our study results indicate that patients of older age, with a history of smoking, diabetes, or high BMI face a significantly higher risk of prolonged drainage time and increased total drainage volume than those without these factors. Additionally, male gender and longer operation times were identified as significant risk factors affecting drainage time. For patients with these conditions, clinicians should carefully monitor hemostasis and bleeding in the perioperative period due to the heightened likelihood of prolonged drainage time and increased drainage volume.

## Data availability statement

The raw data supporting the conclusions of this article will be made available by the authors, without undue reservation.

## Ethics statement

The studies involving humans were approved by the Medical Ethics Committee of Tongji Hospital, Tongji Medical College of Huazhong University of Science and Technology. The studies were conducted in accordance with the local legislation and institutional requirements. Written informed consent for participation in this study was provided by the participants’ legal guardians/next of kin.

## Author contributions

Y-QT: Writing – original draft, Writing – review & editing. XR: Writing – review & editing. Y-SY: Writing – review & editing. JW: Writing – review & editing. XL: Writing – review & editing. Z-HG: Writing – review & editing. X-YZ: Writing – original draft, Writing – review & editing.
