# Supplementary material for: Analysis of risk factors affecting the postoperative drainage after a laparoscopic partial nephrectomy: a retrospective study
Source: Front Med (Lausanne). 2024 Jan 24;11:1327882. doi: 10.3389/fmed.2024.1327882 (PMC10847592; doi:10.3389/fmed.2024.1327882)
Supplement: Supplementary file 1 [file Table_1.docx]

| ﻿Variables | ﻿Mean ± SD (range) | n(%) |
| --- | --- | --- |
| Age (years) | 55.8±14.32 | - |
| Height (cm) | 168.62±8.83 | - |
| Weight (kg) | 74.38±12.26 | - |
| BMI (kg/m^2^) | 27.18±6.22 | - |
| Smoking history  History of alcohol consumption  Family history of RCC | -  -  - | 207(54.7%)  102(26.9%)  5(1.3%) |
| Hypertension | - | 48(12.7%) |
| Diabetes | - | 59(15.8%) |
| Heart Diseases | - | 45(11.9%) |
| Tumor diameter (mm) | 27.33±11.28 | - |
| Tumor Side(left) | - | 252(66.7%) |
| ﻿Preoperative blood protein(g/L) | 66.46±3.76 | - |
| Preoperative APTT (seconds) | 37.35±3.16 | - |
| Preoperative PT (seconds) | 17.09±6.23 | - |
| Preoperative D-dimer (ng/mL) | 0.39±0.36 | - |
| Blood loss during operation(mL) | 96.88±9.73 | - |
| Operation time(min) | 131.29±29.72 | - |
| Time of drainage (day) | 4.05±1.68 | - |
| Total ﻿drainage volume(mL) | 282.83±79.73 | - |

Table 1S. ﻿Demographic and clinical characteristics of male patients undergoing laparoscopic partial nephrectomy (n = 378)

BMI：body mass index; APTT: activated partial thromboplastin time; PT: thrombin time SD: standard error; RCC: renal cell cancer
