# Supplementary material for: Analysis of risk factors affecting the postoperative drainage after a laparoscopic partial nephrectomy: a retrospective study
Source: Front Med (Lausanne). 2024 Jan 24;11:1327882. doi: 10.3389/fmed.2024.1327882 (PMC10847592; doi:10.3389/fmed.2024.1327882)
Supplement: Supplementary file 2 [file Table_2.docx]

|  | Univariable | | |  | | | Multivariable | | |
| --- | --- | --- | --- | --- | --- | --- | --- | --- | --- |
|  | β | SE | p-value | |  | β | | SE | p-value |
| Age | 0.023 | 0.012 | 0.008 | |  | \| 0.007 \| \| --- \| | | 0.018 | 0.010 |
| Smoking history  History of alcohol consumption | 1.021  0.627 | 0.093  0.179 | P<0.001  0.722 | |  | 0.712  - | | 0.088  - | P<0.001  - |
| Hypertension | 0.533 | 0.146 | 0.019 | |  | - | | - | - |
| Diabetes | 0.612 | 0.098 | 0.003 | |  | 0.261 | | 0.086 | 0.041 |
| Heart diseases | 0.142 | 0.139 | 0.402 | |  | - | | - | - |
| Operation time | 0.008 | 0.003 | P<0.001 | |  | 0.006 | | 0.007 | 0.027 |
| Tumor diameter | 0.005 | 0.031 | 0.652 | |  | - | | - | - |
| Tumor side | -0.08 | 0.207 | 0.857 | |  | - | | - | - |
| Preoperative APTT | 0.023 | 0.049 | 0.686 | |  | - | | - | - |
| Preoperative PT | -0.004 | 0.008 | 0.723 | |  | - | | - | - |
| Preoperative D-dimer | 0.128 | 0.078 | 0.471 | |  | - | | - | - |
| Blood loss during operation | 0.009 | 0.012 | 0.368 | |  | - | | - | - |
| ﻿Preoperative blood protein | -0.022 | 0.019 | 0.097 | |  | - | | - | - |
| Height | 0.052 | 0.017 | 0.013 | |  | - | | - | - |
| Weight | 0.058 | 0.009 | 0.007 | |  | - | | - |  |
| BMI | 0.183 | 0.008 | P<0.001 | |  | 0.172 | | 0.059 | 0.032 |

Table 2S. Univariable and multivariable linear regression analysis of factors influencing the time of drainage in males (dependent variable; n =378)

BMI：body mass index; APTT: activated partial thromboplastin time; PT: thrombin time SE: standard error
