# Supplementary material for: Analysis of risk factors affecting the postoperative drainage after a laparoscopic partial nephrectomy: a retrospective study
Source: Front Med (Lausanne). 2024 Jan 24;11:1327882. doi: 10.3389/fmed.2024.1327882 (PMC10847592; doi:10.3389/fmed.2024.1327882)
Supplement: Supplementary file 3 [file Table_3.docx]

|  | Univariable | | |  | | | Multivariable | | |
| --- | --- | --- | --- | --- | --- | --- | --- | --- | --- |
|  | β | SE | p-value | |  | β | | SE | p-value |
| Age | 3.652 | 1.052 | 0.007 | |  | 2.448 | | 1.003 | 0.011 |
| Smoking history  History of alcohol consumption | 160.533  98.063 | 28.472  44.658 | P<0.001  0.803 | |  | 130.231  - | | 29.118  - | P<0.001  - |
| Hypertension | 43.813 | 31.585 | 0.302 | |  | - | | - | - |
| Diabetes | 145.663 | 39.61 | 0.008 | |  | 46.558 | | 29.113 | 0.012 |
| Heart diseases | 5.618 | 30.547 | 0.772 | |  | - | | - | - |
| Operation time | 2.441 | 0.732 | 0.038 | |  |  | | - | - |
| Tumor diameter | 2.227 | 1.447 | 0.588 | |  | - | | - | - |
| Tumor side | 17.438 | 22.313 | 0.804 | |  | - | | - | - |
| Preoperative APTT | 8.203 | 5.335 | 0.417 | |  | - | | - | - |
| Preoperative PT | -1.408 | 2.006 | 0.838 | |  | - | | - | - |
| Preoperative D-dimer | 18.002 | 27.332 | 0.661 | |  | - | | - | - |
| Blood loss during operation | 0.034 | 1.104 | 0.694 | |  | - | | - | - |
| ﻿Preoperative blood protein | -1.002 | 4.305 | 0.586 | |  | - | | - | - |
| Height | 7.406 | 3.412 | 0.007 | |  | - | | - | - |
| Weight | 14.27 | 1.012 | P<0.001 | |  | - | | - |  |
| BMI | 34.002 | 4.112 | P<0.001 | |  | 24.882 | | 13.126 | 0.008 |

Table 3S. Univariable and multivariable linear regression analysis of factors influencing the total volume of drainage in males (dependent variable; n =378)

BMI：body mass index; APTT: activated partial thromboplastin time; PT: thrombin time SE: standard error
