# Supplementary material for: Analysis of risk factors affecting the postoperative drainage after a laparoscopic partial nephrectomy: a retrospective study
Source: Front Med (Lausanne). 2024 Jan 24;11:1327882. doi: 10.3389/fmed.2024.1327882 (PMC10847592; doi:10.3389/fmed.2024.1327882)
Supplement: Supplementary file 4 [file Table_4.docx]

|  | Age | Smoking history | History of alcohol consumption | Hypertension | Diabetes | Heart diseases | Operation time | Tumor diameter | BMI |
| --- | --- | --- | --- | --- | --- | --- | --- | --- | --- |
| Age | 1 | - | - | - | - | - | - | - | - |
| Smoking history | 0.225* | 1 | - | - | - | - | - | - | - |
| History of alcohol consumption | 0.117** | 0.371* | 1 | - | - | - | - | - |  |
| Hypertension | 0.086 | 0.212* | 0.177* | 1 | - | - | - | - | - |
| Diabetes | 0.074 | 0.017 | 0.056 | 0.019 | 1 | - | - | - | - |
| Heart diseases | 0.077 | 0.13 | 0.301 | 0.07 | 0.012 | 1 | - | - | - |
| Operation time | 0.092 | 0.105* | 0.227 | 0.02 | 0.068 | 0.09 | 1 | - | - |
| Tumor diameter | -0.05 | 0.069 | 0.069 | -0.053 | -0.039 | 0.017 | 0.155** | 1 | - |
| BMI | -0.052 | 0.503 | 0.322* | 0.178** | 0.316** | 0.022 | 0.193** | -0.041 | 1 |

Table 4S. Pearson correlation and Spearman’s rank correlation analyses between other studied variables except for time of drainage and total drainage volume in males.

*, correlation is significant at the 0.05 level (two-tailed); **, correlation is significant at the 0.01 level (two-tailed) BMI: body mass index
