# Supplementary material for: Analysis of risk factors affecting the postoperative drainage after a laparoscopic partial nephrectomy: a retrospective study
Source: Front Med (Lausanne). 2024 Jan 24;11:1327882. doi: 10.3389/fmed.2024.1327882 (PMC10847592; doi:10.3389/fmed.2024.1327882)
Supplement: Supplementary file 5 [file Table_5.docx]

| ﻿Variables | ﻿Mean ± SD (range) | n(%) |
| --- | --- | --- |
| Age (years) | 52.8±14.42 | - |
| Height (cm) | 160.41±7.67 | - |
| Weight (kg) | 61.84±14.72 | - |
| BMI (kg/m^2^) | 24.32±7.19 | - |
| Smoking history  History of alcohol consumption  Family history of RCC | -  -  - | 21(8.9%)  28(11.9%)  2(0.9%) |
| Hypertension | - | 21(9.0%) |
| Diabetes | - | 40(17.0%) |
| Heart Diseases | - | 29(12.3%) |
| Tumor diameter (mm) | 27.05±10.14 | - |
| Tumor Side(left) | - | 157(67.1%) |
| ﻿Preoperative blood protein(g/L) | 66.72±4.88 | - |
| Preoperative APTT (seconds) | 34.79±2.97 | - |
| Preoperative PT (seconds) | 16.63±6.07 | - |
| Preoperative D-dimer (ng/mL) | 0.40±0.37 | - |
| Blood loss during operation(mL) | 94.87±10.01 | - |
| Operation time(min) | 128.43±32.13 | - |
| Time of drainage (day) | 3.23±1.33 | - |
| Total ﻿drainage volume(mL) | 242.79±68.57 | - |

Table 5S. ﻿Demographic and clinical characteristics of female patients undergoing laparoscopic partial nephrectomy (n = 234)

BMI：body mass index; APTT: activated partial thromboplastin time; PT: thrombin time SD: standard error; RCC: renal cell cancer
