# Supplementary material for: Analysis of risk factors affecting the postoperative drainage after a laparoscopic partial nephrectomy: a retrospective study
Source: Front Med (Lausanne). 2024 Jan 24;11:1327882. doi: 10.3389/fmed.2024.1327882 (PMC10847592; doi:10.3389/fmed.2024.1327882)
Supplement: Supplementary file 6 [file Table_6.docx]

|  | Univariable | | |  | | | Multivariable | | |
| --- | --- | --- | --- | --- | --- | --- | --- | --- | --- |
|  | β | SE | p-value | |  | β | | SE | p-value |
| Age | 0.019 | 0.003 | 0.028 | |  | \| 0.013 \| \| --- \| | | 0.012 | 0.003 |
| Smoking history  History of alcohol consumption | 0.987  0.542 | 0.086  0.193 | P<0.001  0.785 | |  | 0.502  - | | 0.125  - | P<0.001  - |
| Hypertension | 0.492 | 0.155 | 0.013 | |  | - | | - | - |
| Diabetes | 0.607 | 0.149 | P<0.001 | |  | 0.224 | | 0.063 | 0.019 |
| Heart diseases | 0.157 | 0.196 | 0.501 | |  | - | | - | - |
| Operation time | 0.011 | 0.008 | 0.087 | |  | - | | - | - |
| Tumor diameter | 0.005 | 0.028 | 0.717 | |  | - | | - | - |
| Tumor side | -0.09 | 0.133 | 0.784 | |  | - | | - | - |
| Preoperative APTT | 0.021 | 0.032 | 0.465 | |  | - | | - | - |
| Preoperative PT | -0.003 | 0.016 | 0.658 | |  | - | | - | - |
| Preoperative D-dimer | 0.133 | 0.087 | 0.378 | |  | - | | - | - |
| Blood loss during operation | 0.008 | 0.012 | 0.423 | |  | - | | - | - |
| ﻿Preoperative blood protein | -0.020 | 0.014 | 0.102 | |  | - | | - | - |
| Height | 0.039 | 0.033 | 0.025 | |  | - | | - | - |
| Weight | 0.050 | 0.008 | P<0.001 | |  | - | | - |  |
| BMI | 0.151 | 0.011 | P<0.001 | |  | 0.203 | | 0.052 | 0.016 |

Table 6S. Univariable and multivariable linear regression analysis of factors influencing the time of drainage in females(dependent variable; n =234)

BMI：body mass index; APTT: activated partial thromboplastin time; PT: thrombin time SE: standard error
