# Supplementary material for: Analysis of risk factors affecting the postoperative drainage after a laparoscopic partial nephrectomy: a retrospective study
Source: Front Med (Lausanne). 2024 Jan 24;11:1327882. doi: 10.3389/fmed.2024.1327882 (PMC10847592; doi:10.3389/fmed.2024.1327882)
Supplement: Supplementary file 7 [file Table_7.docx]

|  | Univariable | | |  | | | Multivariable | | |
| --- | --- | --- | --- | --- | --- | --- | --- | --- | --- |
|  | β | SE | p-value | |  | β | | SE | p-value |
| Age | 3.533 | 1.008 | 0.013 | |  | 2.322 | | 0.756 | 0.031 |
| Smoking history  History of alcohol consumption | 154.382  97.003 | 29.754  43.053 | P<0.001  0.778 | |  | 124.658  - | | 30.447  - | P<0.001  - |
| Hypertension | 43.577 | 31.662 | 0.187 | |  | - | | - | - |
| Diabetes | 140.422 | 42.17 | 0.020 | |  | 46.302 | | 22.746 | 0.028 |
| Heart diseases | 5.936 | 31.407 | 0.689 | |  | - | | - | - |
| Operation time | 2.229 | 0.792 | 0.057 | |  | - | | - | - |
| Tumor diameter | 2.206 | 1.401 | 0.560 | |  | - | | - | - |
| Tumor side | 17.286 | 29.885 | 0.601 | |  | - | | - | - |
| Preoperative APTT | 8.220 | 5.469 | 0.512 | |  | - | | - | - |
| Preoperative PT | -1.344 | 1.894 | 0.772 | |  | - | | - | - |
| Preoperative D-dimer | 17.322 | 29.046 | 0.704 | |  | - | | - | - |
| Blood loss during operation | 0.030 | 0.889 | 0.542 | |  | - | | - | - |
| ﻿Preoperative blood protein | -1.042 | 2.973 | 0.508 | |  | - | | - | - |
| Height | 7.293 | 3.526 | 0.023 | |  | - | | - | - |
| Weight | 13.76 | 0.497 | 0.001 | |  | - | | - |  |
| BMI | 31.273 | 4.826 | P<0.001 | |  | 24.242 | | 12.753 | 0.025 |

Table 7S. Univariable and multivariable linear regression analysis of factors influencing the total volume of drainage in females (dependent variable; n =234)

BMI：body mass index; APTT: activated partial thromboplastin time; PT: thrombin time SE: standard error
