# Supplementary material for: Analysis of risk factors affecting the postoperative drainage after a laparoscopic partial nephrectomy: a retrospective study
Source: Front Med (Lausanne). 2024 Jan 24;11:1327882. doi: 10.3389/fmed.2024.1327882 (PMC10847592; doi:10.3389/fmed.2024.1327882)
Supplement: Supplementary file 8 [file Table_8.docx]

|  | Age | Smoking history | History of alcohol consumption | Hypertension | Diabetes | Heart diseases | Operation time | Tumor diameter | BMI |
| --- | --- | --- | --- | --- | --- | --- | --- | --- | --- |
| Age | 1 | - | - | - | - | - | - | - | - |
| Smoking history | 0.407* | 1 | - | - | - | - | - | - | - |
| History of alcohol consumption | 0.238* | 0.329* | 1 | - | - | - | - | - | - |
| Hypertension | 0.069 | 0.262* | 0.192* | 1 | - | - | - | - | - |
| Diabetes | 0.082* | 0.027 | 0.049 | 0.027 | 1 | - | - | - | - |
| Heart diseases | 0.052 | 0.05 | 0.271 | 0.09 | 0.010 | 1 | - | - | - |
| Operation time | 0.081 | 0.088* | 0.389 | 0.05 | 0.091 | 0.10 | 1 | - | - |
| Tumor diameter | -0.12 | 0.074 | 0.077 | -0.044 | -0.037 | 0.027 | 0.153** | 1 | - |
| BMI | -0.058 | 0.469 | 0.202* | 0.172* | 0.297* | 0.042 | 0.188** | -0.032 | 1 |

Table 8S. Pearson correlation and Spearman’s rank correlation analyses between other studied variables except for time of drainage and total drainage volume in females.

*, correlation is significant at the 0.05 level (two-tailed); **, correlation is significant at the 0.01 level (two-tailed) BMI: body mass index
